# A doctor-nurse-patient mobile health management system effectively controls blood glucose in chinese patients with type 2 diabetes mellitus: a prospective study

**DOI:** 10.1186/s12913-022-08949-5

**Published:** 2022-12-21

**Authors:** Xiaoqing Tan, Zonghai Qi, Ling Chen, Dongmin Li, Xiangyin Cai, Yi Song, Yajie Liu

**Affiliations:** 1 Department of Neurology, Shenzhen Hospital, Southern Medical University, NO.1333, Xinhu Road, Baoan District, Shenzhen, Guangdong 518000 China; 2 School of Foreign Studies, Southern Medical University, Guangzhou, Guangdong 510515 China; 3 Nursing Department, Shenzhen Hospital, Southern Medical University, Shenzhen, Guangdong 518000 China; 4 Out-patient Department, Shenzhen Sami International Medical Center, Shenzhen, Guangdong 518118 China; 5 Out-patient Department, Shenzhen Qianhai Free Trade Zone Hospital, Shenzhen, Guangdong 518038 China; 6 Shenzhen Second People’s Hospital, The First Affiliated Hospital of Shenzhen University, Shenzhen, Guangdong 518037 China

**Keywords:** Glycaemic control, Type-2 diabetes mellitus, Blood glucose, Telemedicine, Self-management

## Abstract

**Background:**

Coronavirus-2019 pandemic in China aroused increasing interest in telemedicine-supported glycaemic control. We hypothesize that age might influence usage and efficacy of telemedicine-supported glycaemic control. This study aims to measure the effects of a doctor-nurse-patient Mobile Health Management System (MHMS) for fasting plasma glucose (FPG) control in patients with type 2 diabetes mellitus (T2DM).

**Methods:**

Four hundred sixty four patients with T2DM were recruited. A one-hour diabetes education provided to each patient and subsequent follow-ups arranged in the 1st, 2nd, 4th, 8th, and 12th week after enrollment were recorded in MHMS. The effectiveness of MHMS was defined as the proportion of patients achieving FPG target (below 126 mg/dL or 7.0mml/L).

**Results:**

Among the enrolled 464 patients (age: 55.0 ± 13.7 years) who were divided into three groups: young (18–40 years), middle-aged (41–65 years) and elderly (> 65 years), 424 ones completed all follow-ups of 12 weeks. FPG decreased from 178.38 ± 95.04 to 117.90 ± 14.22 mg/dL in the young group, from 180.00 ± 91.08 to 122.94 ± 37.95 mg/dL in the middle-aged group, and from 174.24 ± 80.64 to 128.88 ± 23.4 mg/dL in the elderly group. The proportion of FPG-target-achieved patients increased from 46.2 to 90.4% in the young group, from 32.6 to 82.8% in the middle-aged group, and from 29.5 to 73.3% in the elderly group. The proportion of FPG-target-achieved patients between three age groups were statistically significant (*P* < 0.001). And the changes of proportion of FPG-target-achieved patients at different follow-up times were statistically significant (*P* = 0.037). Compared with the young group, the elderly group achieved poorer FPG level (*P* = 0.032).

**Conclusion:**

MHMS can help patients with T2DM lower FPG and improve proportion of FPG-target-achieved patients. Younger patients may achieve better glycaemic control than older patients. MHMS may serve multitudinous patients with T2DM to achieve adequate FPG self-management.

**Supplementary Information:**

The online version contains supplementary material available at 10.1186/s12913-022-08949-5.

## Background

Type 2 diabetes mellitus (T2DM) is a leading cause of death among adults. It is estimated that almost 500 million people have been diagnosed with T2DM worldwide [1]. In 2019, there were 116 million diabetic patients living in China, and this number is expected to rise [2]. Poor glycaemic control is associated with high proportion of complications including neuropathy, stroke, and congestive heart failure [3–5]. These complications not only increase morbidity and mortality, but also financially burden patients, families, and society [6]. In the era of the coronavirus-2019 pandemic, telemedicine has become an essential tool for glycaemic control [7], hence it is imperative to evaluate and optimize newly developed telemedicine management systems.

Telemedicine is defined as the remote exchange of medical information and/or services between patients and clinicians through electronic information communication technologies [8, 9]. It has already been used to transmit weekly blood glucose data from patients to clinicians and facilitate follow-up [10, 11]. It has also been used to provide education, support, and cellphone text message reminders to improve the self-management of patients with T2DM.

In 2017, 13,000 medical institutions across 22 Chinese provinces developed a telemedicine system which provided teleconsultation, telediagnosis, and remote medical education. China has a universal health coverage through the social insurance scheme [8]. However, China still has fewer telemedicine users as compared with the United States, Canada, and European countries [9]. Additionally, technological advancements and the coronavirus-2019 pandemic have led to increasing interest in telemedicine for glycaemic monitoring and control [10].

Age is an important factor which may influence both the usage and efficacy of telemedicine-based health management and glycaemic control in patients with T2DM. Others have shown that older patients may be less willing and reluctant to utilize remote health management platforms [11, 12]. Barrot-de la Puente reported that the percentage of patients with fair glycaemic control (HbA1c ≤ 7%) was significantly higher among older aged groups (≥ 65) [13], while Chiu reported that age was negatively correlated with HbA1c [14]. Additionally, there is no independent analysis on the relationship between age, as a single factor, and the management of fasting plasma glucose (FPG) through telemedicine.

The mobile health management system (MHMS) was jointly developed by the Chinese Diabetes Society (CDS) and endocrinologists of Shenzhen Qianhai Free Trade Zone Hospital for T2DM management. It is a telemedicine platform that provides patients with disease education, an endocrinologist hotline, and a repository of patient records including their FPG records .The aim of this study was to examine whether the doctor-nurse-patient MHMS effectively controls FPG in young, middle-aged, and elderly patients with T2DM.

## Methods

### Study design and subjects

Patients with T2DM, who presented to the department of endocrinology of Shenzhen Qianhai Free Trade Zone Hospital between April 2014 and January 2016, were recruited to participate in this prospective study.

The inclusion criteria were: (1) diagnosed as T2DM [15]; (2) age ≥ 18 years; (3) current treatments of basal insulin (involving treatments combining with oral hypoglycemic agents or pre-prandial insulin). The exclusion criteria included: (1) presence of acute diabetic complications, such as ketoacidosis, severe infection, and late-stage heart, liver, or kidney diseases; (2) concurrent administration of pharmaceuticals that interfere with glycaemic control, such as glucocorticoids; and (3) presence of disabilities that prevent self-management.

This study was approved by the Ethical Committee of Shenzhen Qianhai Free Trade Zone Hospital (2022 K-W001). All subjects signed the informed consent.

### Patient and public involvement

All the patients were prescribed basal insulin at discharge. They were informed of the plan to manage their T2DM through MHMS. This management involved scheduled follow-up duration and frequency, self-monitoring glucose during follow-up period, an individualized diet and exercise prescription, reporting FPG records in MHMS, and possible influence deriving from their participation.

### Procedures

Patients with T2DM underwent face-to-face interviews and education before enrollment. Seven telephone follow-ups were scheduled over a 12-week period. And prior to each follow-up, MHMS sent a text message to remind the patients to monitor their blood glucose. MHMS included three modules: (1) diabetes mellitus education, (2) an endocrinologist hotline, and (3) patient records. The diabetes mellitus education module informed patients of both knowledge about insulin and methods of insulin injection, self-monitoring of blood glucose (SMBG), and the follow-up plan of this project. The endocrinologist hotline provided a platform to the involved doctors or nurses to do online follow-ups. The patient records section stored patients’ medical archive, including gender, age, body mass index (BMI), diabetes duration, education and recorded patients’ FPG.

### Follow-up

This project was developed and run by a diabetes specialist team. This team involved two endocrinologists and three advanced practice nurses specializing in diabetes. One of the nurses served as the project leader. The endocrinologists set FPG target and designed the blood glucose management plan. Nurses carried out a one-hour diabetes education for each patient. The contents of education included basic information about insulin and insulin injection methods, methods of SMBG, and an individualized diet and exercise plan. Each patient was offered one blood glucose monitor (Baiankang, Bayer Medical Care Co., LTD, Germany) and adequate blood glucose test strips. Further, the nurses arranged follow-ups in the 1st, 2nd, 4th, 8th, and 12th weeks after enrollment. An endocrinologist gave patients online medication guidance in the 4th and 8th weeks. Patients were instructed to independently monitor their FPG. The data of their FPG and insulin injection doses were input into MHMS by nurses. Table 1 displays the details of the implementation plan.

**Table 1:** Description of the mobile health management system

Timeline	Contents
Study initiation	• Baseline characteristics of patients, such as age, educational level, diabetes duration, BMI, comorbidities, pre-enrollment treatment, initial fasting plasma glucose and HbA1c, were collected. • Patients were offered blood glucose management notebooks and teach them to keep records. • Patients were taught the basics about insulin, blood glucose monitoring, diet, and exercise planning. • Patients received face-to face training on insulin injection and blood glucose monitoring techniques
1st week follow-up	• Follow-up of the patients was carried out by nurses over the phone. The items of follow-up included fasting plasma glucose, insulin dose, medication management, and lifestyle modifications.
2nd week follow-up	• The nurse followed up with patients over the phone. Follow-up items are identical as above. Nurses identified unsettled problems from the previous week which were addressed by the nurses’ guidance or instructions.
4th week follow-up	• Medication guidance was provided by doctors over the phone. The guidance included instructions for the adjustment of insulin dose and frequency of glucose monitoring • Follow-up of the patients was carried out by nurses over the phone. Follow-up items are identical as above. Nurses identified unsettled problems from the previous week which were addressed by the nurses’ guidance or instructions.
8th week follow-up	• Follow-up of the patients was carried out by nurses over the phone. Follow-up items are identical as above. Nurses identified unsettled problems from the previous week which were addressed by the nurses’ guidance or instructions.
12th week follow-up	• Medication guidance was provided by doctors over the phone. The guidance included instructions for the adjustment of insulin dose and frequency of glucose monitoring • Follow-up of the patients was carried out by nurses over the phone. Follow-up items are identical as above. Nurses identified unsettled problems from the previous week which were addressed by the nurses’ guidance or instructions.

### Outcome

The primary outcome was measured FPG. Nurses called patients to do follow-up according to the MHMS timeline and recorded each patient’s updated FPG data into MHMS. The calls were automatically recorded by MHMS in the 1st, 2nd, 4th, 8th, and 12th weeks, and only the calls lasting longer than 120s were recorded as valid data.

The effectiveness of MHMS was defined as the proportion of patients achieving FPG below 126 mg/dL (7.0mml/L) which is indicative of adequate glycaemic control.

### Data collection and definition

The baseline characteristics of patients, including gender, age, educational level, diabetes duration, BMI, reported complications, pre-enrollment treatment plan, and initial FPG and HbA1c data, were recorded in MHMS.

### Statistical analysis

Statistical analysis was conducted using IBM SPSS Statistics for Windows, version 22.0 (IBM, Armonk, NY). Descriptive statistics were used to describe the demographic characteristics of the enrolled patients. Pearson’s chi-squared test was used to compare the demographic characteristics of the enrolled patients. Graph Pad Prism 7.0 (Graph Pad Software, San Diego, CA) was used to plot average FPG level of follow-ups. FPG level of different age groups were analyzed through using two-way repeated measures analyses of variance (ANOVA). The least significant difference test was used for pair-wise comparison between groups (LSD). Generalized linear mixed model (GLMM) was used to compare the proportions of FPG-target-achieved patients in different age groups during different follow-ups. Two-tailed *P* value < 0.05 was considered as statistically significant.

Four hundred sixty four patients’ FPG at baseline were all analyzed. Patients with 5 missed FPG records in follow-ups were excluded. The missed data were abandoned and excluded from the subsequent analysis. Missing data were replaced by using the serial mean. The latter is the most frequently used method to account for arbitrary missing data when less than 5% of data is missing.

## Results

A total of 464 patients were enrolled (mean age: 55.0 ± 13.7 years), and 424 of them completed the study. The 424 patients were divided into a young (18–40 years), a middle-aged (41–65 years) and elderly (> 65 years) groups. Diabetes duration, gender, education level, complication proportion, and treatment plan varied significantly between age groups (*p* > 0.05). Notably, the average diabetes duration was 12.1 ± 6.5 years in the elderly group, 6.4 ± 5.6 years in the middle-aged group, and 2.9 ± 3.5 years in the young group. A total of 24.3% of the elderly patients experienced cardiovascular, cerebrovascular, or other complications, while 8.9% of the middle-aged and 5.0% of the young patients experienced these complications. Moreover, the elderly group was more likely to use oral hypoglycemic agents alone to control blood glucose. Table 2 displays the baseline characteristics of the patients.

**Table 2:** Baseline patient characteristics

Characteristic	Total	Age groups			*P*
		Young (18–40)	Middle-aged (40–65)	Elderly (> 65)	
Sample, n(%)	464	60 (12.9)	293 (63.1)	111 (23.9)	
Age(year), Mean ± SD	55.0 ± 13.7	34.2 ± 5.4	52.08 ± 6.7	73.8 ± 5.8	0.000
BMI(kg/m^2^), Mean ± SD	24.0 ± 3.7	24.9 ± 4.5	24.0 ± 3.6	23.5 ± 3.4	0.112
Diabetes duration(year), Mean ± SD	7.4 ± 6.3	2.9 ± 3.5	6.5 ± 5.6	12.1 ± 6.5	0.001
Gender, n(%)					0.003
Male	289 (62.3)	44 (73.3)	190 (64.8)	55 (49.5)	
Female	175 (37.7)	16 (26.7)	103 (35.2)	56 (50.5)	
Education, n(%)					< 0.001
Primary school and below	30 (6.5)	0 (0.0)	10 (33.3)	20 (66.7)	
Junior high school	148 (31.9)	12 (8.1)	102 (68.9)	34 (23.0)	
Senior high school	140 (30.2)	13 (9.3)	92 (65.7)	35 (25.0)	
Junior college	84 (18.1)	14 (16.7)	57 (67.9)	13 (15.5)	
University graduate and above	62 (13.4)	21 (33.9)	32 (51.6)	9 (14.5)	
Complication, n (%)					0.001
Null	408 (87.9)	57 (95.0)	267 (91.1)	84 (75.7)	
Cardio-cerebrovascular disease	22 (4.7)	1 (1.7)	11 (3.8)	10 (9.0)	
Diabetic nephropathy or Peripheral neuropathy or Diabetic foot	34 (7.3)	2 (3.3)	15 (5.1)	17 (15.3)	
Treatment plan before enrolment, n (%)					0.003
Oral hypoglycemic agents,	308 (66.4)	31 (51.7)	197 (67.2)	80 (72.1)	
Oral hypoglycemic agents, and insulin	111 (23.9)	16 (26.7)	68 (23.2)	27 (24.3)	
Others	45 (9.7)	13 (21.7)	28 (9.6)	4 (3.6)	
FPG at baseline(mmol/L), Mean ± SD	10.1 ± 5.0	10.1 ± 5.1	10.2 ± 5.2	9.8 ± 4.5	0.778
HbA1c at baseline(%), Mean ± SD	9.9 ± 2.6	10.8 ± 2.6	10.0 ± 2.7	9.1 ± 2.3	0.055

*Note*: Values are expressed as number (percentage) or mean ± standard deviation

*Abbreviations*: BMI, body mass index; FPG, fasting plasma glucose

The three main treatment regimens prescribed were 1) basal insulin, 2) basal insulin and oral hypoglycemic agents, and 3) basal insulin and pre-prandial insulin. There were no significant differences in the treatment regimens taken by patients among different age groups after enrollment. Endocrinologists called to follow up the patients twice during the study period. Comparisons of the times of patients’ receiving endocrinologists’ calls between different groups were of no statistical significance (Table S1).

During the 1st follow-up, 15 patients did not answer calling and 25 left wrong numbers. Among the 40 patients, 8 were from the young group, 26 from the middle-aged group, and 6 from the elderly group; 21 are male(52.5%), 19 female (47.5%), and mean age 51.68 + 13.54. There was no significant difference in the ratios of follow-up loss among different age groups (*P* = 0.205). The patients with less than 5 times of FPG data were excluded. Finally 424 patients were included in the final analysis (Figure S1).

**Fig. 1:**
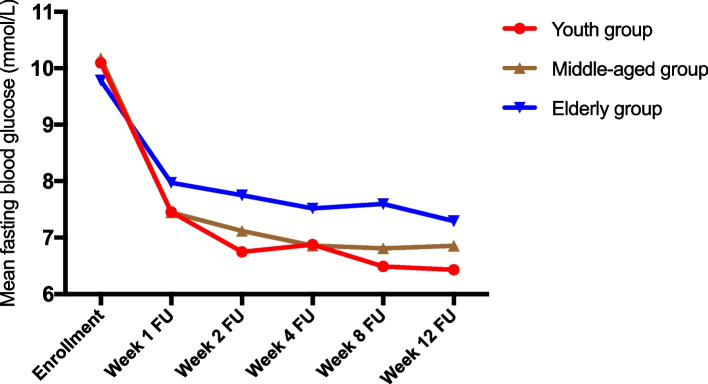
Mean FPG by age group over the course of the study. All age groups experienced a reduction in mean FPG over the course of the study, but young and middle-aged patients responded better than elderly ones

The enrolled patients’ FPG level overall decreased with increase of follow-up visit. FPG of patients in three groups decreased rapidly in the first week of the study and plateaued during later stages (Fig. 1). Decrease of patients’ FPG level in each group was respectively significant (*P* < 0.001), and difference of patients’ FPG level between age groups was significant (*P* < 0.001) (Table 3). Further pair-wise comparison showed that during the study the elderly experienced smaller decrease of blood glucose than the young (*P* = 0.001) and the middle-aged (*P* < 0.001). The young and the middle-aged groups experienced comparable decrease of FPG level (*P* = 0.359) (Table 4).

**Table 3:** Relationship between age and FPG at each follow-up visit

Group	n	FPG (mg/dL), Mean ± SD						*P*-value		
		Baseline	1st week	2nd week	4th week	8th week	12th week	group	time	group*time
Young (18–40)	52	178.38 ± 95.04	134.28 ± 49.32	123.3 ± 28.80	124.20 ± 28.98	118.44 ± 16.38	117.90 ± 14.22	< 0.001	< 0.001	0.862
Middle-aged (40–65)	267	180.00 ± 91.08	134.10 ± 37.26	128.52 ± 34.92	123.66 ± 28.08	122.76 ± 25.02	122.94 ± 19.98			
Elderly (> 65)	105	174.24 ± 80.64	142.38 ± 34.02	138.42 ± 34.2	133.38 ± 29.88	133.02 ± 31.14	128.88 ± 23.4			

**Table 4:** Comparison of the change in FPG over time among young, middle-aged, and elderly patients

Group	Age	Mean difference	95% confidence interval of mean difference		Standard error	*P* value*
			Lower limit	Upper limit		
Young (18–40)	41–65	-0.152	-0.477	0.173	0.165	0.359
	≥ 66	-0.642^*^	-1.005	-0.278	0.185	0.001
Middle-aged (40–65)	≤ 40	0.152	-0.173	0.477	0.165	0.359
	≥ 66	-0.490^*^	-0.737	-0.243	0.126	< 0.001
Elderly (> 65)	≤ 40	0.642^*^	0.278	1.005	0.185	0.001
	41–65	0.490^*^	0.243	0.737	0.126	< 0.001

*Note*: *LSD

The proportions of patients with adequate FPG (≤ 126 mg/dL, or ≤ 7.0 mmol/L) during each follow-up visit are shown in Table 5. The proportion of patients with FPG level below 126 mg/dL increased from 46.2 to 90.4% in the young group, from 32.6 to 82.8% in the middle-aged group, and from 29.5 to 73.3% in the elderly group (Table 5).

**Table 5:** Comparison of the proportion of patients with eligible FPG at different follow-up times

Group	n	Baseline	1st week (%)	2nd week (%)	4th week (%)	8th week (%)	12th week (%)	P time	P group	P time*group
Young (18–40)	52	46.2	57.7	53.8	71.2	84.6	90.4	< 0.001	0.037	0.566
Middle-aged (40–65)	267	32.6	46.8	50.9	74.9	79.0	82.8			
Elderly (> 65)	105	29.5	36.2	39.0	62.9	66.7	73.3^a^			

^a^There was a statistical difference between the older and younger groups (*P* = 0.032)

A generalized mixed linear model was used to compare the proportion of patients achieving adequate FPG between age groups, and the results showed that there were significant differences between three age groups (*P* < 0.001). As the management progressed, the proportion of patients with adequate FPG increased, and this change was statistically significant (*P* < 0.001). Age was a statistically significant factor related to FPG control in this model. There were significantly different proportions of patients achieving adequate FPG in each age group (*P* = 0.037). Compared with the young group, the elderly group had a significant lower percentage of patients with FPG level below 126 mg/dL (*P* = 0.032) (Table 5).

## Discussion

This study demonstrated that MHMS can help all patients with T2DM improve FPG control. In the first week of the study, FPG level decreased dramatically among all patients irrespective of age, and FPG control was improved throughout the intervention via MHMS. However, there was a significantly greater proportion of young and middle-aged patients achieving adequate FPG control as compared with elderly patients.

Two reasons could contribute to patients’ adequate FPG. Firstly, injecting basal insulin after enrollment may be related with patients’ achieving adequate FPG. Secondly, better FPG control may also have been related with patients’ increased self-management ability derived from the education session and regular support through frequent follow-ups. Compared with others’ reports that arranged follow-ups and suggested interventions in 3 or 6 months after patients’ discharge [16], this study exhibits that a better management strategy requires to arrange a follow-up in the first week immediately after patients’ discharge. This follow-up aims to address any problems that can occur during the implementation of the prescribed treatment plan. Then, the subsequent follow-up visits can be conducted in 3 and 6 months to track the patient’s self-management and evaluate their health condition.

This study found that elderly patients with T2DM experienced a less improvement in FPG control from telemedicine management than the young. The elderly are one of the primary target groups for telemedicine program because more than 50% of the elderly are afflicted with multiple chronic conditions which require long-term case management [17]. A Nielson Norman Group study which examined technological capabilities across different age groups found that the elderly had a significantly lower success ratio and a six-fold higher error ratio when completing an assigned task on a computer than young adults [18]. Additionally, most elderly patients prefer face-to-face interactions with family physicians, specialists, and nurses [19].

These findings indicate that patients’ demographic characteristics, learning perspectives, and technological access should be considered when designing and performing chronic disease self-management education programs [20]. It also confirms that age and educational level influence FPG self-management [21, 22]. Consistent with the results of a cross-sectional study [23] and contradictory to those of two retrospective studies [13, 14], there was no significant difference in the proportion of middle-aged and young patients achieving adequate glycaemic control. Although age could lead to worsened glycaemic control among elderly patients [24, 25], this study found that the proportion of elderly patients achieving adequate FPG can significantly increase when the patients received regular follow-ups via MHMS (Table 5).

As a chronic disease, T2DM requires long-term self-management to achieve adequate FPG. The self-management abilities of patients with T2DM can be improved through standardized education and regular follow-up [26–28]. Different from other telemedicine management systems which use text messages or various online applications [29, 30], our endocrinologists and nurses contacted patients directly via MHMS to address their problems and improve their FPG self-management. Moreover, this study demonstrated that MHMS could work as a platform for 2 doctors and 3 nurses to help 424 patients with T2DM control their FPG during the same period. This indicates that MHMS may be applied to serve multitudinous patients with T2DM.

As a prospective study, there are some limitations in this study. First, the recorded FPG depended on patients’ self-monitoring, which might cause some measured deviation. To improve the reliability of data, we offered each enrolled patient a same brand-name blood glucose detector, adequate blood glucose test strips, and an education session. This offer could help patients report valid data of their FPG self-management during the study. Second, this study used FPG rather than HbA1c as evaluating indicator, which is against conventional procedure of favoring HbA1c. However, HbA1c in China is only measured once every three months during treatment initiation and once every 6 months when achieving planned treatment target. However, because of short study duration (3 months) and short intervals between follow-up visits, this study fixed FPG as evaluating indicator. Thirdly, although this study showed that using MHMS could help patients achieve better FPG management in short term, yet the effects of MHMS in long term remained to be further proved.

The study has several strengths. First, we constructed a doctor-nurse-patient team for glycaemic control, emphasizing cooperation between endocrinologists and nursing specialists. So this study was different from conventional follow-up in China which was typically done only by nurses. Second, this study developed a telemedicine-based follow-up system for patients with T2DM. This design provides patients with more comprehensive and systematic instructions of glycaemic control to achieve effective glycaemic control and so can bring greater convenience to patients with T2DM.

## Conclusion

MHMS with regular follow-up and guidance can help patients with T2DM at different ages achieve adequate glycaemic control and lower FPG. Younger patients can achieve better glycemic control than older patients.

This study demonstrates that MHMS may be applied to serve multitudinous patients with T2DM to achieve adequate FPG self-management.

## Supplementary Information

**Additional file 1.**

## Data Availability

All data generated or analysed during this study are included in this published article [and its supplementary information files].

## Abbreviations

MHMS: Mobile Health Management System
FPG: fasting plasma glucose
T2DM: Type 2 diabetes mellitus
CDS: Chinese Diabetes Society
SMBG: self-monitoring of blood glucose
BMI: body mass index
ANOVA: analyses of variance
LSD: least significant difference
GLMM: Generalized linear mixed model
