# Supplementary material for: A doctor-nurse-patient mobile health management system effectively controls blood glucose in chinese patients with type 2 diabetes mellitus: a prospective study
Source: BMC Health Serv Res. 2022 Dec 21;22:1564. doi: 10.1186/s12913-022-08949-5 (PMC9768766; doi:10.1186/s12913-022-08949-5)
Supplement: Supplementary file 1 — Additional file 1. [file 12913_2022_8949_MOESM1_ESM.docx]

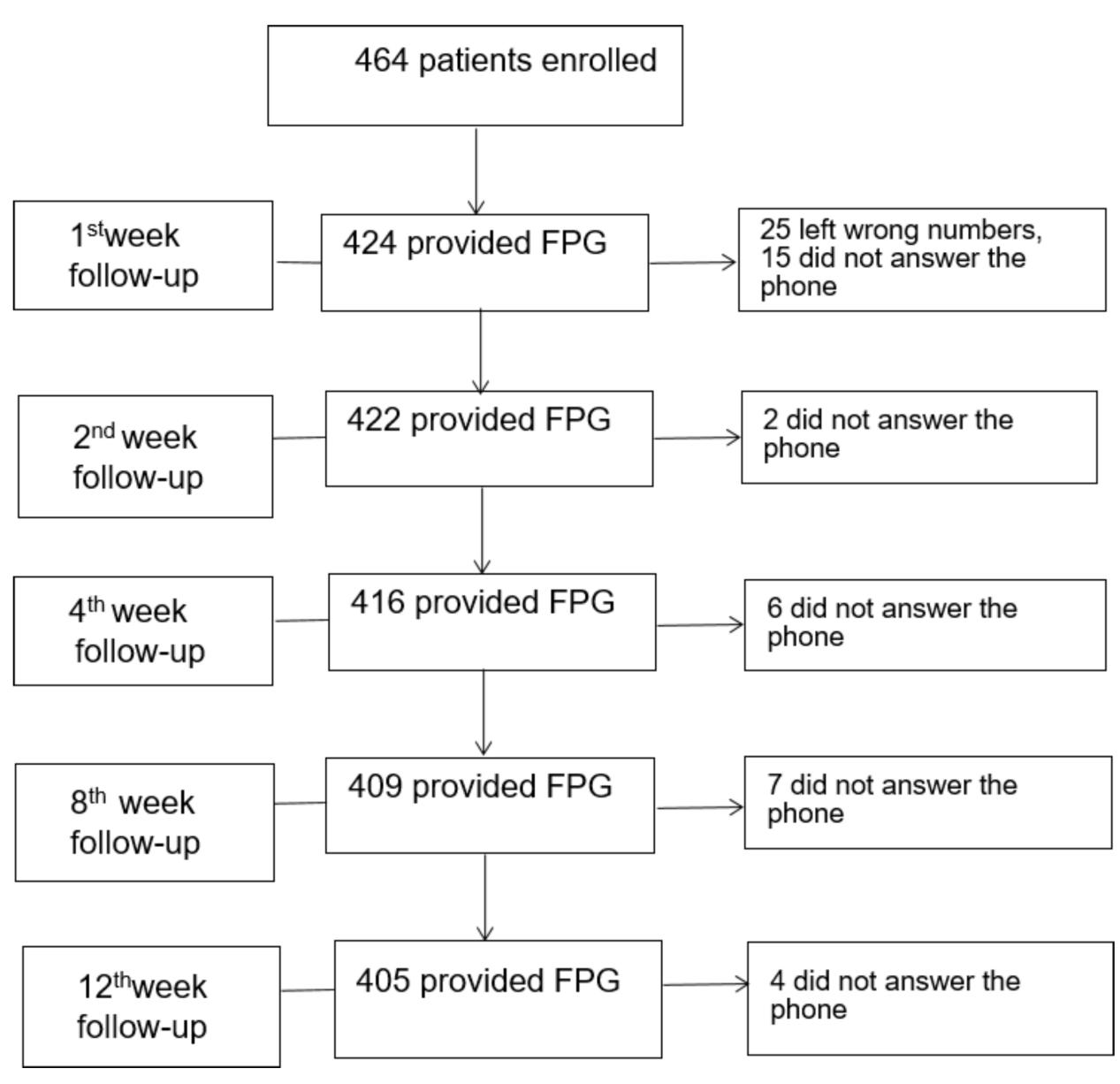


**Figure S1.** Enrollment and retention of study participants


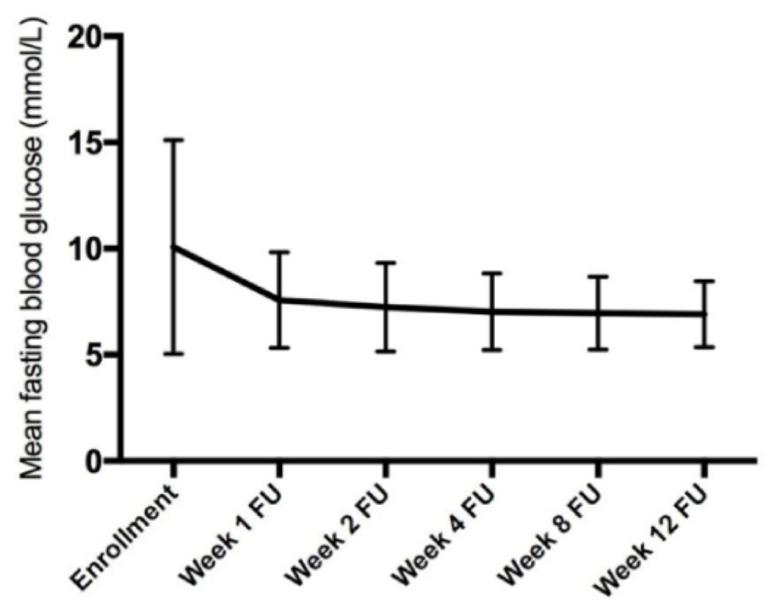


**Figure S2.** Mean FPG at each follow-up visit.

**Table S1 Comparison of treatment plans after enrollment and doctor’s follow-up encounters among different age groups**

| Characteristic | age groups | | | | P |
| --- | --- | --- | --- | --- | --- |
|  | Young (18-40) | middle-aged (40-65) | Elderly (> 65) | |  |
| Sample, n(%) | 60 (12.9) | 293 (63.1) | | 111 (23.9) |  |
| Treatment plan after enrollment, n(%) |  |  | |  | 0.215 |
| Basal insulin and oral hypoglycemic agents | 31 (51.7) | 168 (57.3) | | 74 (66.7) |  |
| Basal insulin and pre-prandial insulin | 27 (45.0) | 108 (36.9) | | 31 (27.9) |  |
| Basal insulin | 2 (3.3) | 17 (5.8) | | 6 (5.4) |  |
| call by doctor(times), Mean±SD | 0.93 ± 1.01 | 1.06 ± 1.02 | | 0.87 ± 1.05 | 0.136 |
